# Observations on Colchicum

**Published:** 1846-12

**Authors:** M. Donovan


*Observations on Colchicum.* By M. Donovan, Esq.—The effects which colchicum produces on the human body are now well ascertained, although the mode of preparation, and the parts of the plant to be preferred, are not yet agreed on. Some prefer the dried bulb, some the recent bulb; one employs the wine of the bulb, another the vinegar of it, another the extract made by evaporating the vinegar: the oxymel has even been a favorite; but the seeds appear to be most generally approved of.

Before the grounds of preference can be understood, it is to be inquired how far the drying of the colchicum bulb interferes with its powers. Analogy tends to render it probable that the efficacy is impaired. Other bulbs, as garlic, onions, leeks, &c., are not only altered by drying, but rendered altogether destitute of these stimulating qualities for which they are valued. Squill, it is true, is not rendered powerless by drying, but its activity is certainly lessened. Dr. A. T. Thomson says—"The acrimony on which its virtue depends is partially dissipated by drying and long keeping, and completely destroyed by any heat above 212 deg." If the bulb of colchicum be injured by drying, how much more so must be the acetous extract, in the preparation of which, unless a steam bath be employed, the heat rises above 212 deg. The vinum colchici of the three British pharmacopœias is made from the dried bulbs, and therefore must be of inferior efficacy.

I believe the most efficacious preparation of the bulb is the wine produced from it in its recent and undried state, as recommended by the late Sir Everard Home, who published three papers on it in the *Philosophical Transactions* for 1816 and 1817. In these papers he has given an account of its preparation, and of its effects, therapeutic and physiological. He directs two pounds of the recent bulbs, undried, to be macerated with twenty-four ounces of sherry wine in a gentle heat for six days.

He convinced himself by repeated trials, that this vinum colchici operates in every respect like the eau medicinale in removing the pains of gout. In his own case the symptoms disappeared in six hours after taking the remedy; but with other persons they did not go off for twelve hours, or even twenty-four. He found that, like the eau medicinale, it diminished the frequency of the pulse 10 or 20 beats in a minute, in twelve hours after taking the dose; and this he considers the criterion by which we may ascertain that the constitution is under its influence.

With regard to the *modus operandi* of colchicum, he conceives that it produces its effects on the circulation, and not on the stomach. This he ascertained in the following manner. Thirty drops of colchicum wine were injected into the circulation, through the jugular vein, of a dog. The pulse increased 40 beats in a minute, and intermitted: in seven hours, he had a motion, and was well. In another experiment, the same dog got a double dose by the jugular, which produced much languor; but he recovered.

He says that the effects on the dog were the same as on himself. In a violent fit of gout, he took sixty drops of eau medicinale, which he considers the same as wine of colchicum. He soon became hot and thirsty; in three hours, the pain was much diminished: in seven hours, nausea came on: his pulse, which was naturally 80, fell to 60, and intermitted: and he became languid; but next day he was quite recovered.

In another experiment Sir E. Home injected 160 drops of colchicum wine into the jugular of a dog: the animal instantly lost all power of voluntary motion; the breathing became slow; and the pulse was scarcely to be felt. In two hours, the pulse rose from 80 to 150. In five hours after, he became very languid, and the pulse was very weak: he vomited some bloody mucus and died. The stomach and duodenum were found in a high state of inflammation. These facts Sir E. Home conceives to prove that the effects are exerted on the circulation, and not on the stomach, in the same way as every poison is known first to enter the circulation, before it specifically affects particular parts.

At the suggestion of Sir E. Home, these experiments on dogs were repeated, with eau medicinale in place of colchicum, by Mr. Gatcombe: and the results were nearly the same; which is a still further evidence of the identity of these two medicines.

The colchicum bulb, Sir E. Home says, contains both extractive and mucilage, both of which, wine, in the first instance, takes up; but when the liquor is strained and allowed to stand, a considerable deposit is almost immediately separated.

This deposit he found to be not only active but virulent; six grains of it given to a dog, by the mouth, produced vomiting and purging which continued for twenty-four hours, the latter evacuations of both kinds being tinged with blood.

Coinciding with the supposed identity of the eau medicinale, which also lets fall a deposit, Sir E. Home concludes, from some experiments, that this remedy, when it contains the deposit suspended in it, produces double the irritation on the stomach and intestines that the clear vinum colchici does. He found that in an instance where he took a dose of eau medicinale, without having shaken the bottle, it was mild in its effects; but that the other half, which contained the deposit, when swallowed, on a different occasion was very severe.

When the deposit is separated from wine of colchicum, he found that it by no means becomes inert. On the contrary, the filtered wine cured a person, on whom it was tried, of a fit of the gout, as well as if it had been in it.

These facts are of great importance, and require to be attended to in the use of this medicine; for we can separate the vomiting and purging portion from that portion which only exerts a specific action on the gout, by removing the deposit from the eau medicinale or colchicum wine according to the conditions of the patient's case; and this is more necessary in the use of the eau medicinale, as its violence has in some cases proved fatal.

The sale of eau medicinale was some years since prohibited in France on account of a nefarious use to which it had been applied.

The deposit is most probably, as Sir E. Home and Mr. Brande suppose, a substance analagous to the deposit which settles from the juice of the wild cucumber named elaterium. This once separated, the juice becomes, like the filtered colchicum wine, mild in its operation.

Sir Joseph Banks, convinced by the evidence contained in these papers of Sir Everard Home, that the vinum colchici, from which the deposit has been removed, must be a less hurtful medicine than the eau medicinale, thought it a duty to himself and the public to make trial of it. When the gout in his left hand and in the joints of that side of the body was very severe, he allowed Sir Everard Home to give him ninety drops of the vinum colchici, and found that the symptoms of gout were sooner and more completely removed than they had ever been by the eau medicinale of which he had experience during seven years, having taken it regularly, and kept a regular account of the doses, their effects, and the intervals between them.

When the variable strength of the different preparations of colchicum, arising from age, climate, soil, season, and manipulation is considered, it becomes a question whether it might not be better to reject them all, and to introduce exclusively into the materia medica the active principle of the plant. Indeed this idea has been already acted on in Italy. Professor Quadri recommends the employment of a proximate principle discovered by him in colchicum, which he calls colchicina, and which he found most useful in gout, and less inconvenient than the bulb.—(*Annali Universali di Medicini da Omodei* 61, 410.] The production of known effects from a known dose would thus be as certainly ensured as in the case of any other medicine. Another advantage would be that inasmuch as the true antarthritic powers of the bulb cannot be always brought to bear on the disease on account of the veratria, which Pelletier and Caventou proved it to contain, we could then increase the dose without any second source of apprehension.

Colchicina possesses great energy. MM. Geiger and Hesse administered one-tenth of a grain to a cat, eight weeks old, which killed the animal in twelve hours, after varied and excessive suffering. The stomach and intestines were found violently inflamed,—(*Journal de Pharmacie*, xx. 164.)

Until this change is made, the best preparation of the bulb is undoubtedly Sir Everard Home's wine, made from the fresh bulb, dug at the end of July, sliced thin, and the slices, as fast as cut, instantly thrown into the wine.

It is a common practice with physicians, in this country, to direct wine of the *seeds* of colchicum in their prescriptions, as if it were officinal in our pharmacopœias. But no such preparation is in them; and the misconception, so very general on this subject, is productive of much uncertainty and inconvenience to the apothecary. The vinum colchici of the pharmacopœias, as already remarked, is made from the dried bulbs ; not from the seeds : from the latter, a tincture is made ; and as it is one of great power there ought to be no confusion connected with it.

I have known the seeds beaten into a mass with mucilage (a work of no small labour) and formed into pills, to act as a brisk cathartic, and to give complete relief in facial neuralgia.

The acetum colchici of the pharmacopœias, neutralized with magnesia, and holding dissolved some sulphate of magnesia, is recommended by Sir C. Scudamore, in his treatise on Gout, as the best formula. He says the combination is inoffensive to the stomach, and certain in its effects on the bowels. The dose recommended by him is half a drachm to one drachm and a half; and this he says never produces constitutional nervousness. I have taken two ounces, however, of the acetum colchici, within six hours, at two equal doses, without any other result than an intermitting pulse. A gentleman labouring under gout, took, on my assurance of the feebleness of this preparation, no less than eighteen drachms of it, in divided doses, every day, for six days, without any obvious effects.

Should Sir Everard Home's wine of colchicum ever come into use, and his authority ought to be sufficient warrant for its introdcction, the apothecary should keep it in two states, one with the deposit, and the other without it.—*Dublin Medical Press.*

